# The prevailing infection of *Schistosoma japonicum* and other zoonotic parasites in bubaline reservoir hosts in the ricefield of lake ecosystem: the case of Lake Mainit, Philippines

**DOI:** 10.1017/S0031182023000537

**Published:** 2023-08

**Authors:** Leonardo A. Estaño, Joycelyn C. Jumawan

**Affiliations:** 1 Department of Biological Sciences, College of Science and Mathematics, Mindanao State University- Iligan Institute of Technology, Iligan City, Philippines; 2 Department of Biology, College of Mathematics and Natural Sciences, Caraga State University, Butuan City, Agusan del Norte, Philippines

**Keywords:** bovines, lakescape, neglected tropical diseases

## Abstract

Bovines are important reservoir hosts of schistosomiasis, placing humans and animals in rice fields areas at risk of infection. This study reported the prevailing infection of zoonotic parasites from bovine feces in the rice fields adjacent to Lake Mainit, Philippines. Formalin Ethyl Acetate Sedimentation was performed on 124 bovine fecal samples from rice fields and documented eggs and cysts from seven parasites: *Schistosoma japonicum, Fasciola gigantica, Ascaris* sp., *Strongyloides* sp., *Balantidium coli,* coccidian oocyst and a hookworm species. Among these parasites, *F. gigantica* harboured the highest infection with a 100% prevalence rate, followed by hookworms (51.61%), *B. coli* (30.64%) and *S. japonicum* (12.09%), respectively. The intensity of infection of *S. japonicum* eggs per gram (MPEG = 4.19) among bovines is categorized as ‘light.’ Bovine contamination index (BCI) calculations revealed that, on average, infected bovines in rice fields excrete 104 750 *S. japonicum* eggs daily. However, across all ricefield stations, bovines were heavily infected with fascioliasis with BCI at 162 700 *F. gigantica* eggs per day. The study reports that apart from the persistent cases of schistosomiasis in the area, bovines in these rice fields are also heavily infected with fascioliasis. The study confirms the critical role of bovines as a reservoir host for continued infection of schistosomiasis, fascioliasis and other diseases in the rice fields of Lake Mainit. Immediate intervention to manage the spread of these diseases in bovines is recommended.

## Graphical abstract

**Figure:**
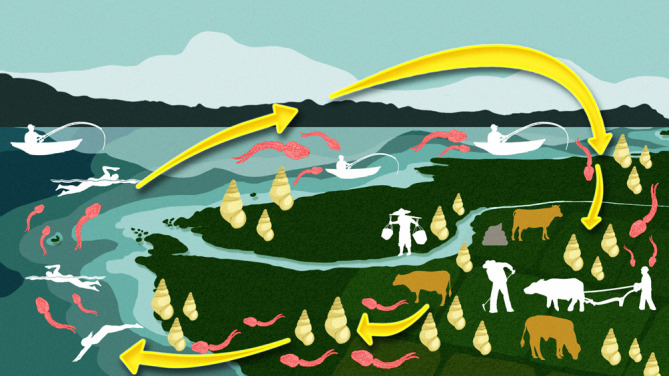

## Introduction

The Philippines is an endemic area of a myriad of neglected tropical diseases (NTDs), six of which: lymphatic filariasis, schistosomiasis, soil transmitted diseases, foodborne trematodiases, rabies and leprosy are of public health importance (Leonardo *et al*., 2020). Schistosomiasis and fascioliasis are among the notable parasitic infection shared by human and bubaline reservoir hosts. In most cases, bovines play a significant role in transmitting parasitic diseases as reservoir hosts that release thousands of parasite eggs daily in the environment, which then develop into larvae or other infective stages (Gordon, 2012; Aragaw and Tilahun, 2019).

There are four municipalities bordering Lake Mainit, most of which have rice fields adjacent to the lake. The lake scape communities surrounding Lake Mainit have been reported endemic for schistosomiasis as early as 1947 and have hampered the lake's tourism and economy in general (Cassion *et al*., 2013). The rice fields strategically located adjacent to Lake Mainit were suitable nidus of active parasite transmission via bubaline reservoir hosts because farming is still mostly unmechanized (Jumawan *et al*., 2020; Jumawan and Estaño, 2021). The lake-rice field interface is often extensively flooded during rainy months, which could promote the spread of zoonotic diseases through bovine fecal matter and snails serving as hosts to several parasitic species (Jumawan *et al*., 2016; Aragaw and Tilahun, 2019). Initial surveys have documented the link between snails and bovines in spreading the disease in ricefields (Jumawan and Estaño, 2021) and other bovine-associated parasitic diseases (Jumawan *et al*., 2020). The occupational risk of farmers and lakeshore residents to schistosomiasis includes exposure to water bodies (irrigated canals, rice paddies, swamps and residential areas) where snails and bovines thrive (Jumawan *et al*., 2016).

The Philippines’ prevention and control of schistosomiasis mainly focused on chemotherapy for human hosts (Leonardo *et al*., 2020). Nonetheless, reports of the critical role of water buffaloes as primary reservoir hosts in spreading the disease have been reported (Gray *et al*., 2008; McManus *et al*., 2011; Gordon *et al*., 2012). The zoonotic nature of the disease calls for a multidisciplinary, multisectoral approach that should engage communities and their leaders, medical professionals, veterinarians, ecologists, malacologists, environmentalists and educators (Tenorio *et al*., 2021). An integrated approach to control the disease should include operational components such as adequate water supply and sanitation, environmental management, snail control, health education, chemotherapy (Praziquantel) and vaccination (Jumawan and Estaño, 2021).

Schistosomiasis and other zoonotic diseases in bubaline reservoir hosts remain largely unknown in the rest of the endemic foci (Tenorio *et al*., 2021). Additional surveys are needed to provide other vital information to raise awareness and proper management of the transmission of pathogenic parasites recovered in bovine feces in the rice fields of these areas. This study reported the updated and consistent prevalence of *S. japonicum, F. gigantica* and other zoonotic parasites in bovine reservoir hosts in the Lake Mainit ecosystem.

## Materials and methods

### Study area

**Figure 1.**
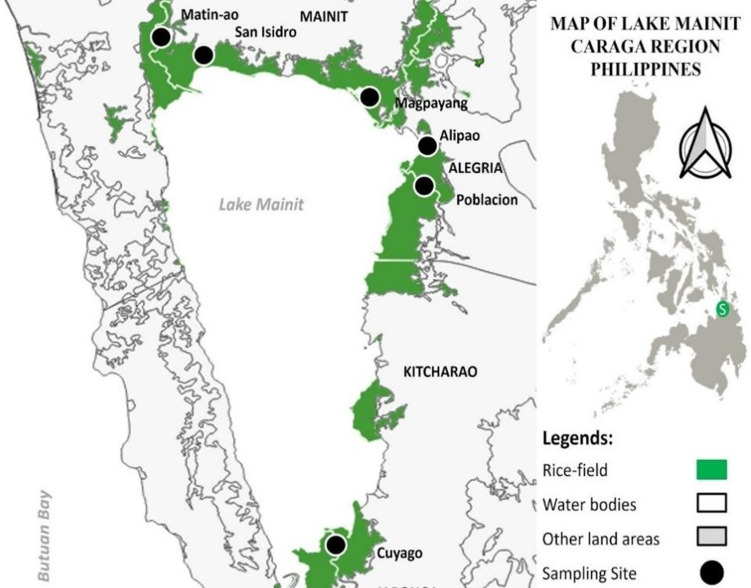
Map of ricefield stations in Lake Mainit, Philippines.

Fecal samples were collected from the rice fields of six lakeside barangays near Lake Mainit, namely the barangay Matin-ao, San Isidro, Alipao, Poblacion Alegria, Magpayang and Cuyago (Fig. 1), from August to November 2021. These shoreline barangays were chosen based on schistosomiasis cases reported from stool data and previous studies in the area (Abao-Paylangco *et al*., 2019; Jumawan *et al*., 2020). Additional sampling stations were also explored aside from the sampling points surveyed by Jumawan and Estaño (2021). A geographical position satellite, model GARMIN GPS 72, was used to take the geographical locations of all sampling sites where fecal samples were collected. The map was constructed using QGIS v.3.22.1 software.

### Collection of fecal samples from bovines

Consent from Local Government Units (LGUs), ricefield and bovine owners was obtained before fecal collection. Collection of feces was done by scooping 3–5 g of freshly fallen bovine feces (at least 24 h since defecation) using a fecal scooper and storing them in sterile containers with 2 mL of 10% formaldehyde for preservation (Jumawan *et al*., 2020; Jumawan and Estaño, 2021). Scooping of samples was obtained from the upper surface of feces to avoid ground contamination. The sex and species origin of the bovine fecal sample source was not determined. Fecal samples were collected from the actual bovines exposed to grazing, foraging and farming activities in the selected ricefield stations with the aid of animal owners to ensure the feces were obtained from all bovines in each sampling site.

### Formalin-ethyl-acetate sedimentation (FEA-sd) technique

The stool parasitological examination technique adopted by Jumawan and Estaño (2021) was used in this study. This procedure utilized the novel copro-parasitological method described by Xu *et al*. (2012) for detecting parasite eggs in bovine fecal samples, the FEA-SD., with a few modifications. A modified McMaster Egg Counting Chamber was used to read the entire volume of the sample (Jumawan and Estaño, 2021).

### Statistical analysis

The parasite infection prevalence in bovines was determined based on parasite eggs/cysts in fecal samples. Egg counts in 5 g of feces were noted. The collected bovine fecal samples among stations were tested for their significant association with parasite infection prevalence using Chi-square independent test. The bovine contamination index (BCI) was determined following Gordon *et al*. (2012), Tenorio and Molina (2020) and Jumawan and Estaño (2021). Statistical computations were performed using Quantitative Parasitology (QP) version 3.0. and SPSS v. 20.0 software.

## Results

### Bovine fecal parasites from rice fields

Laboratory analysis recovered seven major parasites: *Schistoma japonicum, Fasciola gigantica, Ascaris* sp., *Strongyloides* sp., *Balantidium coli*, coccidian oocyst and hookworm species from bovine fecal samples. All collected fecal samples (*N* = 124) were positive for parasite infection (Table 1). The chi-square independent test revealed a significant (*P* = 0.001) difference in infection among recovered parasites, with *F. gigantica* (100%) and hookworms (53.08%) having the highest infection prevalence rates (Table 1). The rice fields of Alipao and Cuyago had the most recovered parasite species; however, fecal samples varied in the parasitic load (Table 2). The liver fluke *F. gigantica* had the highest egg counts among other parasites recorded from bovine fecal samples (Table 2). The present survey reveals fecal samples from the ricefields of Cuyago harbour the highest number of *S. japonicum* eggs with a prevalence of 44%, followed by Alipao (18.3%) and Poblacion, Alegria (9.09%), respectively.

**Table 1.** Prevalence rate of bovine fecal parasites from rice fields adjacent to Lake Mainit, Philippines

Parasites	Magpayang (*n* = 18)		Cuyago (*n* = 25)		San Isidro (*n* = 19)		Pob, Alegria (*n* = 22)		Matin.ao (*n* = 18)		Alipao (*n* = 22)		Total sample *N* = 124	
	Infected samples	Prevalence (%)	Infected samples	Prevalence (%)	Infected samples	Prevalence (%)	Infected samples	Prevalence (%)	Infected samples	Prevalence (%)	Infected samples	Prevalence (%)	Total infected	Over-all prevalence
*Fasciola gigantica*	18	100	25	100	19	100	22	100	18	100	22	100	124	100.00
Hookworm	6	33.33	16	64	7	36.84	14	63.64	5	27.78	16	72.73	64	51.61
Coccidian oocysts	0	0.00	4	16	0	0	0	0	0	0	0	0.00	4	3.23
*Ascaris* sp.	0	0.00	4	16	0	0	0	0	0	0	0	0.00	4	3.23
*Balantidium coli*	3	16.67	7	28	0	0	14	63.64	0	0	14	63.64	38	30.65
*Strongyloides* sp.	0	0.00	0	0	0	0	0	0.00	0	0	3	13.64	3	2.42
*Schistosoma japonicum*	0	0.00	9	36	0	0	2	9.09	0	0	4	18.18	15	12.10

**Table 2.** Eggs/Cyst per count of bovine fecal parasites from rice fields adjacent to Lake Mainit, Philippines

Parasites	Eggs/Cyst per count (EPG/CPC)					
	Magpayang	Cuyago	San-Isidro	Poblacion, Alegria	Matin-ao	Alipao
*Fasciola gigantica*	404	356	134	134	210	168
Hookworm	17	81	8	51	6	42
Coccidian oocysts	0	9	0	0	0	0
*Ascaris* sp.	0	9	0	0	0	0
*Balantidium coli*	2	25	0	31	0	36
*Strongyloides* sp.	0	0	0	0	0	3
*Schistosoma japonicum*	0	53	0	18	0	25

### Multiple parasite infection

This study recorded ten combinations of multiple infections of parasites in various rice fields adjacent to Lake Mainit (Table 3). Co-infection of *F. gigantica* and hookworms was the most prevalent across sampling sites. Fecal samples from the rice fields of Cuyago have the highest infection (84.2% prevalence rate). A combination of four parasite species in one fecal sample from Cuyago was documented: *F. gigantica,* hookworm, coccidian oocyst and *Strongyloides* sp. Multiple infections with three to two parasite species in various sampling sites were also noted (Table 3).

**Table 3.** Prevalence of multiple parasite infections in bovines from ricefields adjacent to Lake Mainit, Philippines

No. of fecal samples examined	Magpayang *n* = 18	Matin-ao *n* = 18	Alipao *n* = 22	San-Isidro *n* = 19	Pob, Alegria *n* = 22	Cuyago *n* = 25
Multiple infections	No. of infected (prevalence)	No. of infected (prevalence)	No. of infected (prevalence)	No. of infected (prevalence)	No. of infected (prevalence)	No. of infected (prevalence)
*Fasciola gigantica* + Hookworm	4(28.8%)	3 (25%)	14(87.5%)	4 (30.8%)	10 (58.8%)	18(84.2%)
*Fasciola gigantica* + *Balantidium coli*	2(14.3%)	0	0	0	11 (64.7%)	7(36.8%)
Hookworm + *Balantidium coli*	0	0	10 (62.5%)	0	0	0
*Fasciola gigantica* + Hookworm + *Balantidium coli*	0	0	0	0	7 (41.2%)	0
*Balantidium coli* + *Schistosoma japonicum*	0	0	2 (12.5%)	0	0	3(15.8%)
*Fasciola gigantica* + *Balantidium coli* + *Schistosoma japonicum*	0	0	2 (12.5%)	0	2 (11.8%)	0
Hookworm + *Balantidium coli* + *Strongyloides* sp.	0	0	2 (12.5%)	0	0	0
*Ascaris* sp. + *Balantidium coli* + *Schistosoma japonicum*	0	0	0	0	0	2(10.5%)
*Fasciola gigantica* + Hookworm + *Ascaris* sp.	0	0	0	0	0	2(10.5%)
*Fasciola gigantica* + hookworm + coccidian oocyst + *Strongyloides* sp.	0	0	0	0	0	1(5.27%)

### Bovine contamination index (BCI) for *Schistosoma* and *Fasciola*

Calculations of the BCI showed that, on average, infected bovines in key rice fields of Lake Mainit could excrete an average of 104, 750 *S. japonicum* eggs as deposited in the environment each day (Table 4). Bovine schistosome infection can be considered ‘light infection’ for Cuyago (2.28 MPEG), Alipao (1.1) and Poblacion, Alegria (0.8). The present survey recorded a higher BCI of approximately 104, 750 *Schistosoma* eggs daily. However, bovines across all rice field stations were heavily infected with fascioliasis with BCI of 162, 700 *Fasciola* eggs per day (Table 5). Co-infection of *F. gigantica* and *S. japonicum* eggs in fecal samples was low (11–12%; Table 3).

**Table 4.** Bovine contamination index (BCI) for *Schistosoma* in ricefields of Lake Mainit calculated using the arithmetic MEPG of the FEA-s.d. data

Sites	Mean EPG	No. of infected	*BCI Overall	*BCI per bovine
Alipao	1.1	4	111 000	27 750
Poblacion, Alegria	0.8	2	40 000	20 000
Cuyago	2.28	11	627 000	57 000
Magpayang	0	0	0	0
San Isidro	0	0	0	0
Matin-ao	0	0	0	0
Overall	4.19	17	1 780 750	104 750

* Calculated using 25 kg as the daily fecal output for bovines (Gordon *et al*., 2012; Tenorio and Molina, 2020).

**Table 5.** Bovine contamination index (BCI) for *Fasciola* in ricefields of Lake Mainit calculated using the arithmetic MEPG of the FEA-s.d. data

Sites	Mean EPG	No. of infected	*BCI Overall	*BCI per bovine
Alipao	7.63	22	419 650	19 075
Poblacion, Alegria	6.09	22	334 950	15 225
Cuyago	14.24	25	890 000	35 600
Magpayang	22.44	18	1 009 800	56 100
San Isidro	7.05	19	334 875	17 625
Matin-ao	7.63	18	343 350	19 075
Overall	10.25	124	3 177 500	162 700

* Calculated using 25 kg as the daily fecal output for bovines (Gordon *et al*., 2012; Tenorio and Molina, 2020).

## Discussion

The ricefield is a crucial habitat for disease transmission when infected snails are present, and farmers utilize these fields unprotected (Jumawan and Estaño, 2021). The high prevalence of infection of *F. gigantica*, a plant-borne trematode, in the feces of bovines from rice fields is consistent with the previous report of Jumawan *et al*. (2020). Fascioliasis infection occurs when a definitive host (humans or cattle) accidentally ingests the parasite by eating raw watercress or other contaminated freshwater plants and the presence of such intermediate snail hosts (Mas-Coma *et al*., 2009; Chang and Flores, 2015; Portugaliza *et al*., 2019).

*Ascaris suum* is a nematode commonly harboured in pigs and cross-infected with bovines (Taylor *et al*., 2016). Acute lung inflammation, stomach distension and discomfort, and intestinal blockage are among the symptoms of *Ascaris* infections in humans. Both *A. lumbricoides* and *A. suum* infection result in abdominal distension, pain and intestinal obstruction (Bokhari, 2021). In the Philippines, ascariasis is associated with strongyloidiasis in other mammalian animals infection. *Strongyloides stercoralis* is the pathologic agent of strongyloidiasis in humans (Baloria *et al*., 2022). In the present survey, eggs of *Strongyloides* sp. were recovered from bovine fecal samples from barangay Alipao. *Strongyloides* spp. is a common intestinal nematode of mammalian hosts that parasitizes the small intestine and can cause diarrhoea and malnutrition, especially in young animals (Jumawan *et al*., 2020).

Hookworm infection from bovine feces was also initially reported in 2020 (Jumawan *et al*., 2020). This parasite inhabiting mammals’ alimentary system results in anaemia caused by the loss of iron and protein in the stomach (Maharana *et al*., 2015). Their transmission and infection in humans and domestic animals are well-documented, making them a significant neglected tropical disease-causing agent affecting both primates and ruminants (Baloria *et al*., 2022).

*Balantidium coli* was recovered from bubaline fecal samples in four barangays: Magpayang, Cuyago, Poblacion Alegria and Alipao. This protozoan is a common intestinal parasite of pigs and a causal agent of balantidiasis in humans, which could be attributed to backyard pig farming in these areas. Human infection is usually an uncommon occurrence caused by cyst contamination in food and water. These issues are more frequent among malnourished people, those who work with pigs, cattle and other animals, and those who work in unsanitary conditions (Kumar *et al*., 2016). Coccidia is a common intestinal parasite of pigs. Infection in livestock results in weight loss and diarrhoea and affects animal production (Tumusiime *et al*., 2020; Gong *et al*., 2021). This parasite can be a causal agent of coccidiosis, potentially infecting humans (Knight *et al*., 2018).

Incidences of multiple infections, such as *F. gigantica,* hookworm, coccidia and *Strongyloides* sp., in the feces of bovines, were previously reported (Jumawan *et al*., 2020). Bovine fecal samples in the area recovered with *Schistosoma* eggs in Barangay Cuyago and Alipao (Jumawan and Estaño, 2021). Other parasites were also consistently recovered, particularly *Strongyloides* sp., *Ascaris* sp., coccidian oocysts and eggs of hookworm helminths. The current study updates recorded new combinations of multiple infections of intestinal parasites and observed higher prevalence rates of infection. The coccidian oocyst, a common avian parasite (Sood *et al*., 2017), is consistently recovered in fecal samples collected in the ricefields of barangay Alipao, an ecotone interface of wild animals, including migratory birds, bovines and other livestock animals such as ducts, pigs and other ruminants. Emergence and cross-infection of zoonotic parasites in this habitat may take place.

The prevailing infection of *Schistosoma* in bovine fecal samples in the rice fields of Cuyago and Alipao shows a persistent zoonotic transmission in the area (Jumawan and Estaño (2021). *Oncomelania* snails in the ricefields of Alipao harboured schistosome cercaria. In Cuyago, infected snails were found distantly from the ricefields utilized by bovines for bathing and foraging, suggesting that the ricefield is not the only nidus for schistosomiasis emergence (Jumawan and Estaño, 2021).

The earliest case of schistosomiasis in Lake Mainit was reported in 1947 by Pesigan (1947), and the occurrence has been persistently documented from random surveys of human stool samples ever since. The topographic features of Lake Mainit are suitable endemic foci where critical elements for continuous transmission are maintained (Jumawan and Estaño, 2021). The disease is considered a prevailing endemic public health concern that is endemic to Caraga and 11 other regions in the Philippines (Olveda *et al*., 2014; Leonardo *et al*., 2016). The ricefield is a crucial habitat for human schistosomiasis transmission when infected snails are present, and farmers utilize these unprotected fields. Potential high-risk exposure of humans to *Schistosoma* may still be possible even if bovines are absent in rice paddies and other wet areas. Infection can still occur with or without the bovine reservoir host if *Oncomelania* harbouring *Schistosoma* is present.

The survey recorded a higher BCI of approximately 104, 750 *Schistosoma* eggs daily compared to the previous study, with ~ 40, 000 *S. japonicum* eggs in the environment (Jumawan and Estaño, 2021). The increased number of BCI per individual bovines supports the claim of the previous result that the timing of the *Schistosoma* life cycles and egg release in the stool of bovines may have a seasonal variation. The parasite's life cycle may still prevail since *Schistosoma* may utilize other mammalian hosts, such as rodents, dogs, pigs and other nearby ruminants. This factor is considered an alarming eyeshot of uninterrupted transmission of Schistosomiasis in endemic foci. The extensive surveys in other wet areas, as recorded in Cuyago, bovines had the highest infection rate, proving that rice fields may be one of many sources of infection for bovines. However, areas such as those for animal grazing and resting may be potential venues for bovine schistosomiasis (Jumawan and Estaño, 2021). The current survey updated rice fields with infected bovines, particularly Poblacion Alegria. These results demonstrate that bovine zoonosis could be widespread that may serve as a source of parasites capable of infecting humans.

Fascioliasis in the Philippines has been documented most typically through bovines (Gray *et al*., 2008; Mas-Coma *et al*., 2009; Portugaliza *et al*., 2019) but rarely in humans (Gray *et al*., 2008) where they occur due to the consumption of raw water vegetables infested with *Fasciola*. Culturally rooted eating behaviours and sanitation practices in endemic areas are important risk factors for acquiring and perpetuating foodborne trematodiasis, as in the case of fascioliasis (Tenorio and Molina, 2021). While there are two *Fasciola* species in the Philippines, our current study reports the presence of *F. gigantica* (130–145 *μ*m × 70–90 *μ*m). Reports on human fascioliasis in the country are scarce and are primarily random research undertaken by undergraduate and graduate students (Leonardo *et al*., 2020). Bovine monitoring surveys by line agencies of Agusan del Norte and Surigao del Norte do not include the occurrence of schistosomiasis and fascioliasis. Some sections of Mindanao practice building bovine enclosures away from rice fields and storing and drying bovine feces before using them as fertilizer, significantly reducing schistosomiasis cases. They could also be adapted for controlling fascioliasis (Gray *et al*., 2008).

Most recovered parasitic helminths identified in the present study are classified as NTDs causing agents. The high infection of bovine fascioliasis exemplifies that topographic feature favours the zoonosis of parasitic helminth in the ricefield of the lake ecosystem as in the case of Lake Mainit. The lake-ricefield interface may facilitate the synergistic infection of other parasites, such as hookworms, *Strongyloides* sp., coccidian oocysts and *Ascaris* sp., harbouring in bovines and must be given attention for control measures of the transmission to animals and humans. Molecular-based analysis, such as environmental DNA studies, may provide additional data for detecting schistosomiasis and other bovine-mediated diseases.

## Conclusion

The study provided updates on the infection of bubaline reservoir hosts in rice fields adjacent to Lake Mainit by surveying eggs and cysts of parasites from bovine feces. The significant incidence of multiple infections in fecal samples confirms the critical role of bovines as a reservoir host for schistosomiasis and other diseases in the rice fields adjacent Lake Mainit. The current study suggests conducting more research and molecular-based analysis to ensure the sensitivity and efficacy of the bubaline parasitic detection and to explore the potential zoonotic capacity of the recovered parasites. The newly auxiliary positive sites illustrate the prevailing zoonosis transmission in the lake ecosystem and call for urgent health-related interventions such as agricultural practices and environmental modification, bovine vaccination and deworming, and other integrated approaches to control and eradicate zoonotic disease transmission by bubaline reservoir hosts.

## Data Availability

Not applicable.
